# Vulnerabilitäten bei Radiomics: Warum die populärste Radiomics-Signatur unbemerkt das Tumorvolumen gemessen hat

**DOI:** 10.1007/s00066-021-01747-8

**Published:** 2021-02-03

**Authors:** Florian Putz, Rainer Fietkau

**Affiliations:** Department of Radiation Oncology, Universitätsklinikum Erlangen, Friedrich-Alexander-Universität Erlangen-Nürnberg, Universitätsstraße 27, 91054 Erlangen, Deutschland

## Hintergrund und Ziel der Arbeit

In den letzten Jahren wurde eine exponentiell zunehmende Zahl von Radiomics-Arbeiten veröffentlicht. Zu den bekanntesten zählt die grundlegende und mittlerweile mehr als tausendmal zitierte Arbeit von Aerts et al.[1], die mithilfe einer Radiomics-Signatur auf Basis von CT-Bilddaten das Gesamtüberleben (OS) von Patienten mit NSCLC und Kopf-Hals-Karzinomen vorhersagen konnte und in den Folgejahren von nicht weniger als drei weiteren Arbeiten extern validiert wurde. Die Aerts-Radiomics-Signatur wurde nun von Welch et al. über 4 Jahre nach deren Erstbeschreibung erneut unter die Lupe genommen.

## Methode

Die Autoren haben die prognostische Vorhersagekraft der Aerts-Radiomics-Signatur im ursprünglichen NSCLC-Trainingsdatensatz (*n* = 422) und einem Kopf-Hals-Karzinom-Datensatz (*n* = 527) erneut nachvollzogen. Dabei verwendeten sie sowohl die Originalbilddaten als auch Bilddaten mit zufälliger Voxelanordnung, um den Stellenwert der Texturanalyse zu überprüfen. Zusätzlich verglichen sie die prognostische Aussagekraft der Aerts-Radiomics-Signatur kritisch mit dem Tumorvolumen.

## Ergebnisse

Die Radiomics-Signatur zeigte eine signifikante prognostische Vorhersagekraft für das OS. Überraschenderweise ergab sich jedoch kein Unterschied in der Vorhersagekraft, ob die originalen CT-Bilddaten oder Bilder mit zufälliger Voxelanordnung für die Extraktion der Textur-Features verwendet wurden. Auch hatte die Radiomics-Signatur keine bessere Vorhersagekraft als das Tumorvolumen allein. In weitergehenden Analysen zeigten die Autoren, dass 3 von 4 Radiomics-Features und damit auch die gesamte Signatur hochgradig mit dem Tumorvolumen korrelierten (Spearman-Rho 0,76–0,99).

## Schlussfolgerung der Autoren

Die populäre Radiomics-Signatur ist nur ein Surrogat für das Tumorvolumen und die vordergründig gemessenen Intensitäts- und Textureigenschaften der Tumoren sind nicht relevant für die beobachtete prognostische Vorhersagekraft der Radiomics-Signatur.

## Kommentar

Radiomics beschreibt typischerweise die Extraktion hunderter vordefinierter quantitativer Parameter, sog. Features, aus Segmentierungen in medizinischen Schnittbilddaten, die anschließend mittels statistischer und Maschinenlernverfahren für Vorhersagen genutzt werden. Die extrahierten Features werden meistens in die vier Kategorien Intensität, Tumorform und Tumortextur sowie sog. Wavelet-Features, die nach vorangegangener Wavelet-Filterung entstehen, eingeteilt [1]. Mithilfe von Radiomics wurden beindruckende prognostische Vorhersagen, aber auch Rückschlüsse auf Molekularpathologie und molekularbiologische Pathway-Aktivierungen in Tumoren auf der Basis von segmentierten CT- und MRT-Schnittbilddaten beschrieben, die weit über die Möglichkeiten konventioneller menschlicher Betrachtung hinausgingen. Radiomics wird daher als Schlüsseltechnologie für die personalisierte Onkologie gesehen, um aus medizinischen Bilddaten therapierelevante Einblicke in die zugrunde liegende räumlich heterogene Tumorbiologie mit besserer Repräsentativität und höherer zeitlicher Auflösung zu ermöglichen, als dies mit wiederholten Biopsien möglich ist [1–3]. Radiomics löste daher großen Enthusiasmus und eine jährlich steigende Anzahl an Publikationen aus, nicht zuletzt auch in der Radioonkologie.

Besondere Beachtung und Einfluss hatte dabei sicherlich die grundlegende Arbeit von Aerts et al. „Decoding tumour phenotype by noninvasive imaging using a quantitative radiomics approach“, welche 2014 in *Nature Communications* veröffentlicht und mittlerweile über tausendmal zitiert wurde. In dieser Landmark-Studie reduzierten Aerts et al. zunächst 440 primär extrahierte Radiomics-Features auf eine Radiomics-Signatur oder eine Auswahl von lediglich 4 Features, welche jeweils nach Stabilität und prognostischer Vorhersagekraft aus den 4 Grundkategorien Intensitäts‑, Form‑, Textur- und Wavelet-Features ausgewählt wurden. Die ausgewählten 4 Radiomics-Features Energie (Intensität), Kompaktheit (Form), „grey level nonuniformity“ (Textur) und der HLH-Wavelet-Abkömmling wurden dann in einem Trainingsdatensatz aus 422 NSCLC-CT-Datensätzen mit einem einfachen Cox-Regressions-Modell auf das Gesamtüberleben gefittet. Das Radiomics-Modell konnten Aerts et al. in einem externen NSCLC-Datensatz, aber auch in zwei externen Kopf-Hals-Tumor-Datensätzen validieren. Mit einem C‑Index (0,5 zufällige Vorhersage, 1,0 perfekte Vorhersage) von 0,65 bis 0,69 hat die Radiomics-Signatur dabei in allen 3 externen Datensätzen aus Radboud, Maastro und Amsterdam eine gute und mit einem *p* << 0,001 auch hochsignifikante prognostische Vorhersagekraft gezeigt [1]. In den Folgejahren konnte dann die Aerts-Radiomics-Signatur sogar an 3 weiteren externen Datensätzen, u. a. aus Kanada, aber auch an einem Datensatz von 293 deutschen Kopf-Hals-Tumor-Patienten extern validiert werden [3–5]. Gerade diese sehr gute Generalisierbarkeit der Aerts-Radiomics-Signatur über CT-Scanner-Modelle und Kohorten hinweg war vielversprechend und wurde als Indiz für das „translationale Potenzial“ von Radiomics betrachtet, muss jedoch angesichts der hier zu besprechenden Ergebnisse in einem anderen Licht gesehen werden [1].

Über vier Jahre nach Erstveröffentlichung haben Welch und Koautoren nun die Aerts-Radiomics-Signatur mit einer in *Radiotherapy & Oncology* veröffentlichten Arbeit erneut überprüft. Die Schlussfolgerung der Autoren ist dabei sowohl spektakulär als auch ernüchternd zugleich und kann wie folgt zusammengefasst werden: Die populäre Radiomics-Signatur korrelierte in ihrer Auswertung hochgradig mit dem Tumorvolumen, und jede prognostische Vorhersagekraft leitete sich aus dieser zugrunde liegenden Abhängigkeit ab, während die vordergründig gemessenen Intensitäts- und Textureigenschaften keine prognostische Relevanz besaßen.

Welch et al. stützen ihre Schlussfolgerung auf mehrere Analysen. Im Zentrum steht die Anwendung der Aerts-Radiomics-Signatur auf voxelpermutierte CT-Bilder, in denen alle Voxel zufällig verteilt wurden und keinerlei Texturinformationen mehr vorhanden waren. Erstaunlicherweise beobachten sie, dass die prognostische Aussagekraft der Signatur in den Datensätzen mit zufälliger Voxelanordnung nicht geringer war als in den ursprünglichen CT-Datensätzen mit erhaltener Texturinformation (Kopf-Hals-Validierungsdatensatz C‑Index 0,64 vs. 0,64). Wichtig ist, dass für diese Analysen zwar die Voxelintensitäten in den CT-Bilddaten zufällig permutiert wurden, die Tumorsegmentierungen und damit die Tumorvolumeninformationen jedoch beibehalten wurden. Des Weiteren verglichen Welch et al. die prognostische Aussagekraft der Radiomics-Signatur mit der des Tumorvolumens allein und beobachteten dabei keinen Unterschied (*p* = 0,90). Dafür zeigte sich eine hochgradige Korrelation von 3 der 4 Radiomics-Features aus der Aerts-Signatur mit dem Tumorvolumen, und zwar sowohl im originalen NSCLC-Trainingsdatensatz als auch im Kopf-Hals-Validierungsdatensatz (Spearman-Rho 0,76–0,99). Am eklatantesten war diese Korrelation für das Textur-Feature „grey level nonuniformity“ (Rho 0,97 bzw. 0,99) und dessen Wavelet-Abkömmling (Rho jeweils 0,99; [6]). Bereits 2017 hatten Vallieres et al. eine hochgradige (lineare) Korrelation des Radiomics-Feature aus der Aerts-Signatur mit dem Tumorvolumen beschrieben [7]. Nach Volumennormalisierung reduzierte sich erwartungsgemäß die Volumenkorrelation, aber mit ihr auch die prognostische Aussagekraft der Features in der Signatur [6]. Lediglich das Form-Feature Kompaktheit, das die Kompaktheit eines Tumors im Verhältnis zu einer Kugel beschreibt, korrelierte wenig mit dem Tumorvolumen (Rho −0,38) und zeigte dann auch in einem Cox-Regressionsmodell neben dem Tumorvolumen eine unabhängige prognostische Aussagekraft. Zusätzlich zu der zugrunde liegenden Abhängigkeit der Radiomics-Signatur vom Tumorvolumen beobachteten die Autoren außerdem eine hochgradige Multikollinearität zwischen dem Textur-Feature „grey level nonuniformity“ und dessen Wavelet-Abkömmling, was zu Problemen mit der Cox-Modellierung führen kann und zeigt, dass die beiden Features redundant waren.

Die spannendere Frage ist jedoch: Warum wiesen der Großteil der Features und die resultierende Radiomics-Signatur eine so große Abhängigkeit vom Tumorvolumen auf? Hierzu muss man sowohl die Feature-Definition als auch den Prozess der Feature-Selektion in der Originalarbeit von Aerts et al. betrachten. Das Intensitäts-Feature Energie z. B. war definiert als die Summe der quadrierten Intensitäten aller Voxel innerhalb der Tumorsegmentierung und damit direkt von der Anzahl der Tumorvoxel und dem Tumorvolumen abhängig, aber auch der Voxelauflösung sowie der Schichtdicke.

Wie Welch et al. bemerken, hat vermutlich gerade der Prozess der Feature-Reduktion in der Arbeit von Aerts et al., bei der die stabilsten und prognostischsten Features ausgewählt wurden, dazu geführt, dass in erster Linie Features mit zugrunde liegender, aber unbemerkter Volumenabhängigkeit selektioniert wurden, während andere Features entweder in den durchgeführten Test-Retest- oder Mehrfach-Konturier-Auswertungen zu instabil waren oder im NSCLC-Trainingsdatensatz keine ausreichende prognostische Aussagekraft hatten [6].

In einigen Punkten muss man jedoch die Schlussfolgerung der Autoren einschränken. Denn Aerts et al. haben in der Originalarbeit in der Tat die prognostische Aussagekraft der Radiomics-Signatur in drei externen Datensätzen gegenüber dem Tumorvolumen und dem TNM-Stadium verglichen und jeweils auch eine signifikante, zusätzliche prognostische Aussagekraft der Radiomics-Signatur zeigen können. Wobei diese zugegebenermaßen eher moderat ausfiel: (C-Index Radiomics + Tumorvolumen vs. Tumorvolumen allein: 0,65 vs. 0,63; 0,69 vs. 0,68 und 0,68 vs. 0,65). Außerdem haben Welch et al. zwar denselben NSCLC-Trainingsdatensatz wie in der Originalpublikation von Aerts et al. verwendet, der Kopf-Hals-Validierungsdatensatz stammte jedoch aus der Arbeit von Leijenaar et al. [5, 6]. Darüber hinaus erfolgte die Feature-Extraktion in der Arbeit von Welch et al. mittels der Open-Source-Software PyRadiomics, während Aerts et al. einen In-House-Matlab-Code verwendeten, was zu abweichenden Feature-Werten geführt haben wird.

Die Notwendigkeit zur kritischen Auseinandersetzung, Reproduzierbarkeit und Replizierbarkeit von wissenschaftlichen Ergebnissen sollte in keinster Weise die wissenschaftliche Leistung von Aerts und Mitarbeitern schmälern. Im Gegenteil: Es ist sehr zu begrüßen, dass die Gruppe einen Großteil der verwendeten Daten sowie die umfassende Dokumentation zur verwendeten Methodik veröffentlich haben.

Vor allem in den letzten Jahren wurden erhebliche Anstrengungen unternommen, um das Potenzial von Radiomics in die Klinik zu übertragen. Die Image Biomarker Standardization Initiative (IBSI) hat erfolgreich 169 Radiomics-Features unter Beteiligung von 25 Radiomics-Arbeitsgruppen standardisiert und gleichzeitig deutlich gemacht, dass ein großer Bedarf hierfür besteht [8]. Außerdem steht mittlerweile Open-Source-Software wie PyRadiomics zur Feature-Extraktion zur Verfügung, deren Verwendung empfohlen wird [6].

Jedoch hat auch das Streben nach einer weiteren Verbesserung der Methode stellenweise erhebliche Limitationen in den Veröffentlichungen zu Radiomics zutage gebracht. Fornacon-Wood et al. [9] hatten bei 70 % der identifizierten NSCLC-Radiomics-Arbeiten ≥ 6 methodische Einschränkungen identifiziert bei einem medianen Radiomics Quality Score (RQS) von 6 (mögliche Spannbreite von −8 bis 36). Die Tatsache, dass 36 % der untersuchten Arbeiten lediglich eine interne und vor allem 13 % gar keine Validierung durchgeführt haben, muss vermutlich als Indiz dafür gesehen werden, dass noch ein allgemein unzureichendes Bewusstsein für das „overfitting“ von Maschinenlernmodellen oder „validation data leakage“ auch bei Peer-Reviewern und Editoren bestand [9, 10].

Eine Vielzahl von möglichen Einflussfaktoren kann die Ergebnisse von Radiomics-Studien beeinflussen. Diese reichen von der Bildakquisition über die Bildrekonstruktion und -segmentierung bis hin zu Feature-Extraktion und Modellierung. Reproduktion und Replikation der Arbeiten sind nur möglich, wenn genannte Parameter mit veröffentlicht werden [2, 10]. Die IBSI veröffentlichte eine 76 Punkte umfassende „reporting guideline“, die die erforderlichen Parameter auflistet. Darüber hinaus sind die Qualität und Limitationen von Radiomics-Arbeiten schwer für Gutachter, Editoren und Leser einzuschätzen. Scoring-Systeme wie der Radiomics Quality Score (RQS) von Lambin et al. [2], die methodische Limitationsliste von Fornacon-Wood et al. [9], aber auch der Tripod-Score zum Validierungsgrad einer Studie können bei einer umfassenden Bewertung von Radiomics-Arbeiten helfen [2, 9, 10]. Fachzeitschriften könnten die Veröffentlichung der „IBSI reporting guideline“ oder die Selbstbewertung anhand publizierter Scoring-Systeme bei Einreichung von Radiomics-Arbeiten einfordern, um die Qualität und Transparenz veröffentlichter Arbeiten zu verbessern. Darüber hinaus zeigt nicht zuletzt das Beispiel der populären Radiomics-Signatur von Aerts et al. den hohen Stellenwert der Mitveröffentlichung von Bilddaten für die Reproduktion von Radiomics-Arbeiten und die Weiterentwicklung des Gebiets.

### Fazit

Bei erneuter detaillierter Begutachtung einer populären, bereits mehrfach validierten Radiomics-Signatur hat sich erstaunlicherweise gezeigt, dass diese eine hochgradige Abhängigkeit vom Tumorvolumen aufwies und diese unbeabsichtigte Erfassung des Tumorvolumens zumindest für einen Großteil der beobachteten prognostischen Vorhersagekraft verantwortlich war. Radiomics-Modelle müssen eine zusätzliche prognostische Aussagekraft im Vergleich zu den akzeptierten Prognosefaktoren aufweisen und auf Konfundierung mit diesen untersucht werden. Nicht zuletzt hierfür ist die Integration der medizinisch-ärztlichen Expertise erforderlich.

Für die Bewertung von Radiomics-Arbeiten stehen mehrere Scoring-Systeme zur Verfügung. Wichtig ist vor allem, auf die Validierung der Modelle und die detaillierte Beschreibung der zahlreichen möglichen Einflussfaktoren zu achten, z. B. anhand der „IBSI reporting guideline“. Zur Reproduktion von Ergebnissen ist die zusätzliche Veröffentlichung der verwendeten Bilddaten und der Programmcodes sehr zu begrüßen.

*Florian Putz und Rainer Fietkau, Erlangen*
